# Effects of 7.5% Carbon Dioxide and Nicotine Administration on Latent Inhibition

**DOI:** 10.3389/fpsyt.2021.582745

**Published:** 2021-04-16

**Authors:** Kiri T. Granger, Jennifer Ferrar, Sheryl Caswell, Mark Haselgrove, Paula M. Moran, Angela Attwood, Jennifer H. Barnett

**Affiliations:** ^1^Cambridge Cognition, Cambridge, United Kingdom; ^2^School of Psychology, University of Nottingham, Nottingham, United Kingdom; ^3^Monument Therapeutics, Cambridge, United Kingdom; ^4^Alcohol & Tobacco Research Group, University of Bristol, Bristol, United Kingdom; ^5^Department of Psychiatry, University of Cambridge, Cambridge, United Kingdom

**Keywords:** schizophrenia, biomarker, latent inhibition, carbon dioxide challenge, nicotine

## Abstract

Stratified medicine approaches have potential to improve the efficacy of drug development for schizophrenia and other psychiatric conditions, as they have for oncology. Latent inhibition is a candidate biomarker as it demonstrates differential sensitivity to key symptoms and neurobiological abnormalities associated with schizophrenia. The aims of this research were to evaluate whether a novel latent inhibition task that is not confounded by alternative learning effects such as learned irrelevance, is sensitive to (1) an in-direct model relevant to psychosis [using 7.5% carbon dioxide (CO_2_) inhalations to induce dopamine release *via* somatic anxiety] and (2) a pro-cognitive pharmacological manipulation (*via* nicotine administration) for the treatment of cognitive impairment associated with schizophrenia. Experiment 1 used a 7.5% CO_2_ challenge as a model of anxiety-induced dopamine release to evaluate the sensitivity of latent inhibition during CO_2_ gas inhalation, compared to the inhalation of medical air. Experiment 2 examined the effect of 2 mg nicotine administration vs. placebo on latent inhibition to evaluate its sensitivity to a potential pro-cognitive drug treatment. Inhalation of 7.5% CO_2_ raised self-report and physiological measures of anxiety and impaired latent inhibition, relative to a medical air control; whereas administration of 2 mg nicotine, demonstrated increased latent inhibition relative to placebo control. Here, two complementary experimental studies suggest latent inhibition is modified by manipulations that are relevant to the detection and treatment of schizophrenia. These results suggest that this latent inhibition task merits further investigation in the context of neurobiological sub-groups suitable for novel treatment strategies.

## Introduction

The biological heterogeneity of schizophrenia continues to be a major obstacle for clinical practice and the development of novel drug treatments. A non-invasive biomarker to define sub-groups of patients with common neurobiological underpinnings would improve detection, diagnosis and the efficacy of drug development. Abnormal attention is a core deficit of schizophrenia that is commonly modeled pre-clinically using a latent inhibition paradigm (1–4) which may have potential in this regard. In latent inhibition, a stimulus is rendered irrelevant by mere exposure, before being established as a cue for an outcome. Latent inhibition is observed when participants learn more slowly about the preexposed cue than a non-preexposed control cue during a subsequent test of learning (5). Theoretical analyses of latent inhibition have focused upon an attentional explanation—proposing that during preexposure, attention diminishes to the preexposed stimulus so that, subsequently, participants take longer to learn the association between this stimulus and the outcome than the non-preexposed cue (6–8).

Disrupted latent inhibition is widely observed in schizophrenia [for a review see (9)] and can happen in two distinct ways: (1) *An attenuation of latent inhibition*, in which the difference in the rate of learning to the preexposed and non-preexposed stimuli is reduced (and we posit a disrupted ability to reduce attention to the preexposed/irrelevant stimulus). (2) *An enhancement of latent inhibition* in which the difference in the rate of learning to the preexposed and non-preexposed cues is increased (and we posit an enhanced ability to reduce attention to the preexposed/irrelevant stimulus). Latent inhibition thus provides a measure of the balance between these two extremes of attentional processing, which together, are thought to underpin the key symptoms of schizophrenia (4, 10). Attenuated latent inhibition is deemed particularly relevant to the positive symptoms (i.e., hyper-dopaminergic state) of the disorder; with an inability to reduce attention to irrelevant information driving a psychotic state. Whereas enhanced latent inhibition is related to the negative and cognitive symptoms [i.e., cholinergic and hypo-glutamatergic; see (11)]; where augmented reduction in attention to the preexposed stimulus is considered a reflection of an inability to switch attentional responding and learn that the preexposed stimulus is now a predictor of an outcome (9).

In line with the well-known dopaminergic contribution to psychosis (12, 13), rats treated with amphetamine show an attenuation of latent inhibition (14, 15) which is successfully reversed by dopamine-blocking antipsychotic drugs [for a review see (10)]. This has been replicated in humans [see (10, 11)], providing support for amphetamine-induced disrupted latent inhibition as a model of positive symptoms of schizophrenia. In contrast to dopaminergic effects, and in line with the idea that glutamatergic and cholinergic signaling drives the negative and cognitive symptoms of schizophrenia, NMDA antagonists (i.e., MK801) that inhibits glutamate as well as nicotinic acetylcholine receptors (nAChRs) (16) have demonstrated an opposing effect, producing enhancement (excess) of latent inhibition in humans and animals [(10); but see (17)].

The existence of dissociable forms of perturbation in latent inhibition is supported by observations of attenuated latent inhibition in acutely psychotic patients experiencing positive symptoms [e.g., (18–21)], and an enhancement of latent inhibition demonstrated in patients experiencing a predominance of negative and cognitive symptoms (9, 20, 22–24). As these attentional manifestations can be mapped onto underlying neural systems considered dysfunctional in schizophrenia, latent inhibition lends itself as a potential tool for detecting patients with different neurochemical states and symptomologies.

As anti-psychotic treatments are largely ineffective at treating the negative and cognitive symptoms of schizophrenia (25–27), many attempts have been made to develop non-dopaminergic treatments for cognitive impairment associated with schizophrenia. Several of these efforts have emphasized the α7 subtype of nAChRs due to the preponderance of patients with schizophrenia who self-medicate with nicotine to manage cognitive and negative symptoms and the side effects of anti-psychotic medications [(28), but see (29)]. This hypothesis is built on evidence that nicotinic receptor signaling is fundamentally decreased in individuals experiencing schizophrenia, and thus patients are using the most readily available method for pharmacologically targeting this system in an attempt to restore signaling to appropriate levels (30).

In humans, reports of the effects of nicotine on latent inhibition are however limited. Thornton et al. (31) reported that nicotine failed to affect latent inhibition in non-smokers who were tested following subcutaneous administration of nicotine, vs. a placebo-treated control group. Although, in a group of smokers vs. non-smokers, Della Casa and Feldon (32) reported that latent inhibition was enhanced. Pre-clinically however, a α7-nAChR partial agonist, SSR180711, has been shown to reinstate latent inhibition following administration of the NMDA receptor antagonist MK801 (33), as well as improve attention and memory performance. Furthermore, α7-nAChR agonists have been shown to improve P50 attentional gating deficits as well as cognitive performance on measures of sustained attention, measured by the Cambridge Neuropsychological Test Automated Battery (CANTAB) in patients with chronic schizophrenia (34). Additional evidence supports a moderate correlation between P50 and latent inhibition [r > 0.6 (35)]. With the pro-cognitive potential of nicotine-enhancing agents for the treatment of cognitive impairment associated with schizophrenia, the current study aimed to investigate the sensitivity of a novel latent inhibition task [see (36)] to nicotine exposure vs. placebo in non-smoking individuals. Treatment of improved attentional filtering (enhanced latent inhibition) following nicotine vs. placebo treatment could provide evidence to determine the future research and potential clinical validation of this latent inhibition task that may serve as a potential tool to identify patients with schizophrenia most likely to benefit cognitively from a nicotinic-based treatment.

This study aimed to evaluate the sensitivity of latent inhibition to both clinically-relevant (dopaminergic) and pro-cognitive pharmacological (nicotinic) manipulations. Experiment 1 explored the sensitivity of the latent inhibition task to a 7.5% carbon dioxide (CO_2_) challenge as a model of anxiety-induced dopamine release. Given evidence that the 7.5% CO_2_ challenge is accepted as a robust method to induce state anxiety (37, 38) and state anxiety increases dopamine release (39, 40), it was hypothesized that latent inhibition would be attenuated during the CO_2_ gas inhalation, compared to inhalation of medical air, in a single-blind crossover design in healthy volunteers. Experiment 2 conversely explored the sensitivity of the latent inhibition task to a pro-cognitive model relevant to the treatment of cognitive impairment associated with schizophrenia by examining the effect of nicotine administration on latent inhibition. It was hypothesized that latent inhibition would be increased (i.e., improved attentional filtering) following nicotine administration, compared to placebo, in a single-blind crossover design in non-smoking healthy volunteers.

## Experiment 1: Effects of 7.5% Carbon Dioxide Inhalation on Latent Inhibition

### Materials and Methods

#### Design

In experiment 1, 30 healthy volunteers were administered either 7.5% CO_2_ or medical air to induce dopamine release *via* induction of state anxiety, in a single-blind crossover design, with 30-min washout between gas inhalations. The gas orders were counterbalanced across participants.

#### Participants

Thirty non-smoking healthy volunteers were recruited from the University of Bristol and the local community *via* email lists, poster, and fllier advertisements and the Tobacco and Alcohol Research Group newsletter and website. The exclusion criteria were age under 18 or over 50 years, daily smoking, history of drug/alcohol dependency, pregnancy or breast feeding, recent use of prescribed or illicit drugs, uncorrected visual or hearing problems, diagnosed medical illness, and not being registered with a general practitioner. Pregnancy and recent drug use were assessed by urine screen, whereas all other criteria were confirmed by self-report. Participants were also excluded if they had high systolic or diastolic blood pressure (SBP/DBP) (<140/90 mmHg), bradycardia or tachycardia (<50 or >90 beats per min), or a body mass index (BMI) outside a healthy range (<18 or >30 kg/m^2^) (all physically assessed). Psychiatric health was assessed using a truncated MINI International Neuropsychiatric Interview (41). Participants refrained from consuming alcohol for 36 h prior to the study day. Expired breath alcohol and carbon monoxide readings were taken, and participants were to be excluded if the readings were >0 or ≥10, respectively. No candidate participants had to be excluded from the research. The study was approved by the University of Bristol, Faculty of Science Research Ethics Committee. Sample size was determined based on a previous study of a similar nature (38).

#### Gases and Questionnaires

The gases were 7.5% CO_2_ or medical air (21% oxygen; BOC Ltd.). These were administered using an oro-nasal mask (Hans Rudolph, Kansas City, MO, USA). Questionnaires included the State-Trait Inventory for Cognitive and Somatic Anxiety (STICSA) (42), Positive and Negative Affect Schedule (PANAS) (43), and the Oxford-Liverpool Inventory of Feelings and Experiences as a measure of schizotypy to ensure baseline schizotypy scores were within normative range [O-LIFE (44)].

#### Latent Inhibition Task

**Figure 1:**
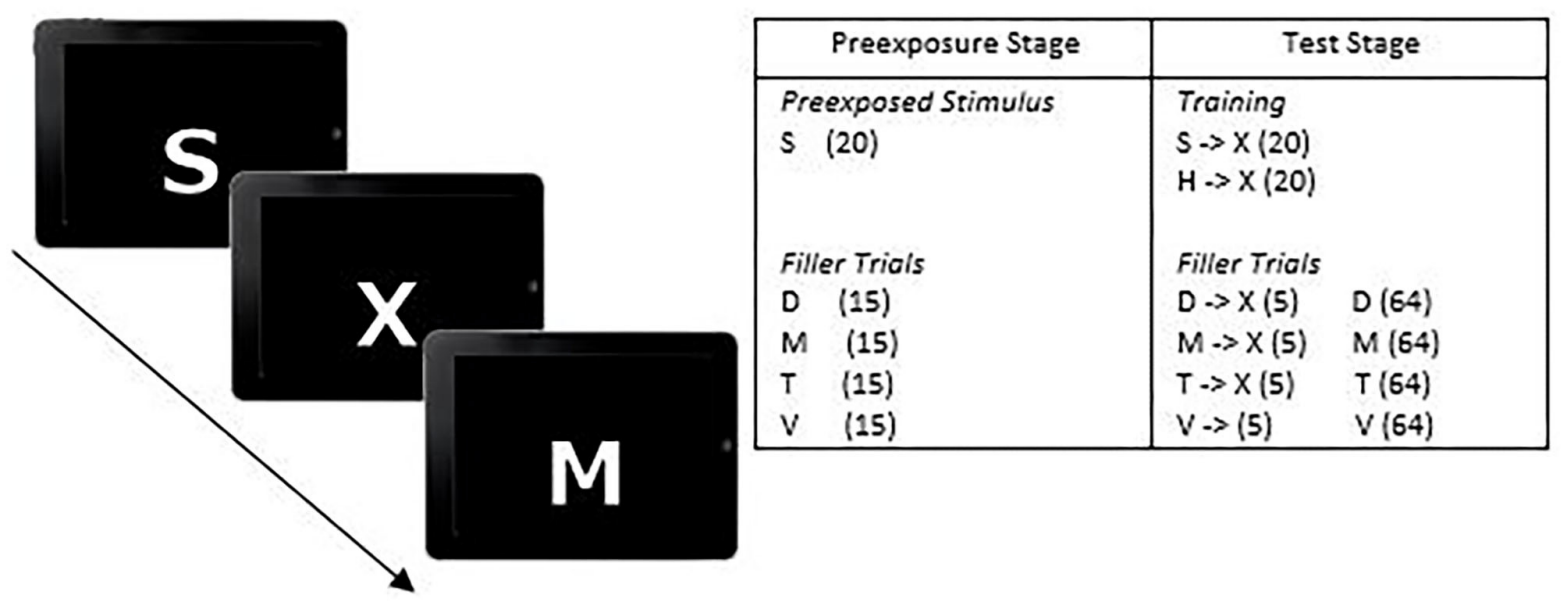
Experimental design and example stimuli for the test stage of the latent inhibition task. Each trial comprised a 1,000 ms presentation of a stimulus separated by an inter-stimulus interval (ISI) of 150 ms. Participants were required to use the computer mouse to click the button on-screen either when the target stimulus “X” appeared on screen, or before it appeared if they could predict it as the next letter in the sequence. The preexposed (PE) and non-preexposed (NPE) stimuli were counterbalanced across participants. Numbers in parentheses in the insert refer to trial frequencies.

A modification of Granger et al.'s (36) latent inhibition task was used and delivered *via* the CANTAB Connect web-based software platform. Two equivalent versions of the task were used (one during each gas inhalation). Each participant completed the task on a 17-in. LCD monitor at a resolution of 1,280 × 1,024 with a 60-Hz refresh rate. The latent inhibition task was accessed *via* a web-based link that directed participants to the CANTAB Connect platform-hosting site for the task and data collection. Stimuli were white capital-letters in Arial-font (7 mm × 5 mm; h × w) presented for 1,000 ms each on the computer-screen with a black background. There were two versions of the task to enable repeat testing that were counterbalanced across participants. For version 1, the stimulus-letters were S and H; one of the letters served as the preexposed stimulus and the other was the non-preexposed stimulus, counterbalanced across participants. The target was the letter X, with filler-letters D, M, T, and V; see Figure 1 for an example. For version 2, the stimulus letters were R and O, and again one of the letters served as the preexposed stimulus and the other was the non-preexposed stimulus, counterbalanced across participants. The target was the letter Z, with filler-letters F, N, K, and A.

Each version of the task had two stages: Preexposure and Test. After reading an information sheet and signing a consent form, the following instructions were presented to participants on the computer monitor prior to the task:

“*In this task you will see a sequence of letters appearing on the screen. Your task is to press the response button at the bottom of the screen each time the current letter is the same as the one that was presented before last, which is 2 positions back in the sequence. Otherwise, do not respond. When this task ends, you will be given a new set of instructions. Press the arrow below when you are ready to begin.”*

During the preexposure stage the preexposed stimulus was presented 20 times, intermixed in a random order with presentations of filler letters each of which was presented 15 times; each stimulus was presented for 1,000 ms separated by a 150 ms inter-stimulus interval. The non-preexposed stimulus and target letter (X or Z) were not presented during the preexposure stage. Following completion of the pre-exposure phase, participants were presenting with a new set of instructions prior to the test phase:

“*In this task you will see a sequence of letters appearing on the screen. Your task is to try and predict when a letter 'X' is going to appear. If you think you know when the 'X' will appear then you can press the response button early in the sequence, which is before the 'X' appears on screen. Alternatively, if you are unable to do this please press the response button as quickly as possible when you see the letter 'X'. There may be more than one rule that predicts the 'X'. Please try to be as accurate as you can, but do not worry about making the occasional error. If you understand the task, please press the arrow below when you are ready to begin.”*

The test stage instructions were the same for the second version of the latent inhibition task but with the instruction to predict the letter “Z” rather than “X.” For the test stage, the preexposed stimulus and the non-preexposed stimulus were each presented 20 times followed by a 1,000 ms presentation of the target stimulus. There were also 20 non-cued presentations of either “X” or “Z” during which the target was preceded by one of the four filler letters, each of which preceding the target five times. In total there were 64 presentations of the filler letters throughout the test phase. The whole task lasted 7 min.

Reaction times (RTs) in the test stage were recorded from the onset of the preexposed and non-preexposed stimulus that preceded the target letter (X or Z) for each participant. Each stimulus was presented for 1,000 ms separated by a 150 ms inter-stimulus interval. Reaction times could range from 0 to 2,150 ms; reaction times <1,150 ms, indicated participants predicted the occurrence of the target as the next letter in the sequence. Whereas, reaction times between 1,150 and 2,150 ms, indicated participants responded to the target when it appeared on screen. Median reaction times for responses to the preexposed and non-preexposed stimuli were calculated for each participant as the median is less biased by extreme values compared to the mean. Correct responses were also calculated for each individual. If the participant had predicted the target (i.e., they had pressed the spacebar on the letter immediately preceding the target) it was deemed that this was a correct response. For each participant the number of correct responses to the preexposed and non-preexposed stimuli were counted separately for each stimulus type (preexposed and non-preexposed).

#### Procedure

Prior to the session, a telephone screen assessed basic eligibility. Eligible participants attended a single test session, at which full written informed consent was obtained and further screening assessments were conducted. If eligibility was met, baseline questionnaire (STICSA, PANAS, and O-LIFE) and cardiovascular [blood pressure (BP) and heart rate (HR)] measures were recorded. The inhalation began with 60 s of free breathing before the tasks were started (this allowed for the gas to start taking effect before data collection began). Inhalations then continued for the duration of the latent inhibition task (up to 20 min for each inhalation). Immediately after each inhalation, measures of BP, HR, STICSA, and PANAS were completed, and there was a 30-min washout period between gas inhalations. The second inhalation followed the same procedure as the first. After the inhalations were complete, participants remained in the room for a minimum of 20 min, to allow any effects to dissipate. Participants were then debriefed and reimbursed £20. A follow-up call was conducted 24 h later to assess whether any adverse events had occurred.

### Results

#### Characteristics of Participants

The participants (*n* = 18; 60% female) were between 19 and 32 years of age (M = 23, SD = 3.4). STICSA state and trait baseline scores ranged between 21 and 50 (M = 28, SD = 7) and between 2 and 31 (M = 25, SD = 5), respectively. Baseline PANAS scores ranged between 21 and 43 (M = 25, SD = 5) and for the sub-dimensions of O-LIFE: Unusual Experiences (positive schizotypy); 0 and 19 (M = 4, SD = 5), Cognitive Disorganization; 0 and 18 (M = 7, SD = 6), Introvertive Anedonia (negative schizotypy); 1 and 11 (M = 4, SD = 3) Impulsive Non-conformity; 0 and 11 (M = 6, SD = 2). O-LIFE scores were relatively comparable to normative values and those reported in previous studies (44) demonstrating baseline schizotypy scores representative of a healthy sample.

#### Subjective and Cardiovascular Effects

State anxiety (STICSA), negative affect (PANAS-negative), SBP, DBP, and HR were higher, and positive affect (PANAS-positive) was lower, after CO_2_ than after medical air inhalation (see Table 1), confirming the validity of the manipulation to induce state anxiety. Importantly, at baseline, there were no significant differences between conditions (CO_2_ vs. medical air) for any subjective or cardiovascular event using independent sample *t*-tests (all *p* > 0.45).

**Table 1:** State anxiety, affect, and cardiovascular function show significant differences during CO_2_ vs. air inhalation (paired *t*-test comparisons).

	**Mean difference (SD): CO_**2**_ vs. air**	**Effect size (Cohen's d)**	***df***	**95% CI**	***p*-value**
STICSA state	10.33 (11.11)	0.95	29	−6.18 to −14.48	0.001
PANAS-positive	−5.23 (4.92)	0.67	29	7.07–3.39	0.001
PANAS-negative	2.73 (3.76)	0.55	29	−1.33 to −4.13	0.001
Systolic BP	9.77 (10.67)	0.75	29	−5.79 to −13.75	0.001
Diastolic BP	1.60 (4.11)	0.18	29	−0.07 to −3.14	0.041
Heart rate	8.27 (10.57)	0.64	29	−4.32 to −12.21	0.001

*STICSA, State-Trait Inventory for Cognitive and Somatic Anxiety; PANAS, Positive and Negative Affect Schedule; SBP, systolic blood pressure; DBP, diastolic blood pressure; HR, heart rate*.

#### Latent Inhibition: Reaction Time

**Figure 2:**
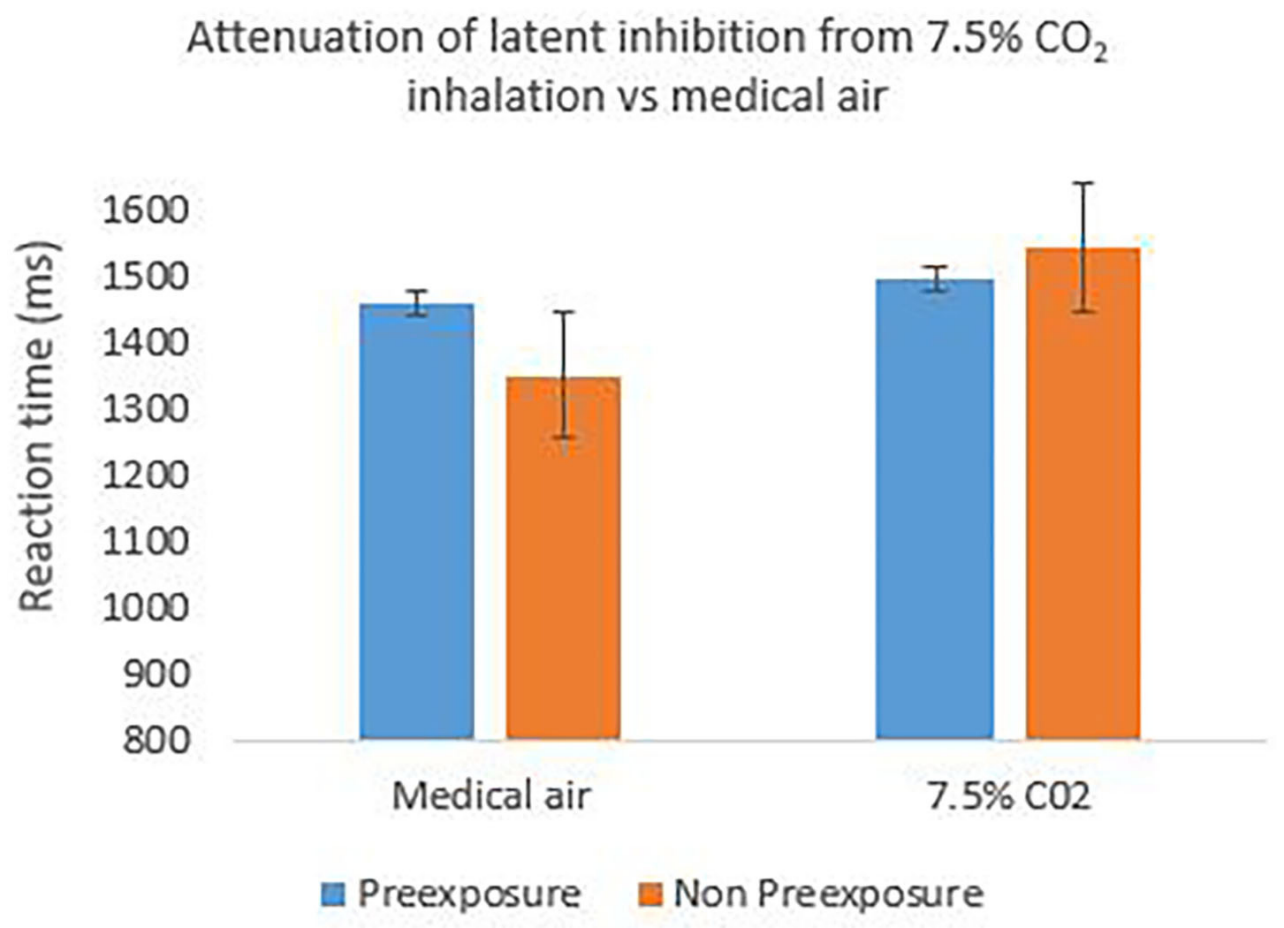
The mean reaction time to the target cued by preexposed stimuli and non-preexposed stimuli in the test stage of the latent inhibition task. Successful effect of latent inhibition is seen in the medical air condition, but attenuated or reversed during CO_2_ inhalation. Error bars are 1± within-subject standard error of the mean [see (45)].

Figure 2 shows the group mean of individual median reaction times to the target (X or Z) across the 20 test trials for the preexposed and non-preexposed stimuli. For the medical air condition, it can be seen that reaction times were slightly faster during the non-preexposed than the non-preexposed stimulus trials, indicating successful induction of the expected latent inhibition effect. In the CO_2_ condition however, the effect is, if anything, in the reverse direction indicating slightly faster reaction times to the preexposed stimulus. This impression was explored using a 2 (stimulus: preexposed, non-preexposed) × 2 (gas: CO_2_, medical air) repeated measures analysis of variance (ANOVA) on individual median reaction times, which revealed a significant main effect of stimulus *F*_(1, 29)_ = 7.718, *p* = 0.009, partial η^2^ = 0.210 indicating an overall effect of latent inhibition; there was no significant main effect of gas (*F* < 1). Pre-planned comparisons revealed a significant effect of stimulus in the medical air *F*_(1, 29)_ = 8.440, *p* = 0.0017 partial η^2^ = 0.225 but not the CO_2_ condition *F*_(1, 29)_ = 1.875, *p* = 0.181; confirming an effect of latent inhibition observable in the anticipated direction in the medical air condition, and an absence of this effect, in the CO_2_ condition, see Figure 2. The overall 2-way interaction (stimulus × gas) was however not significant (*F* < 1).

#### Latent Inhibition: Correct Responses

**Figure 3:**
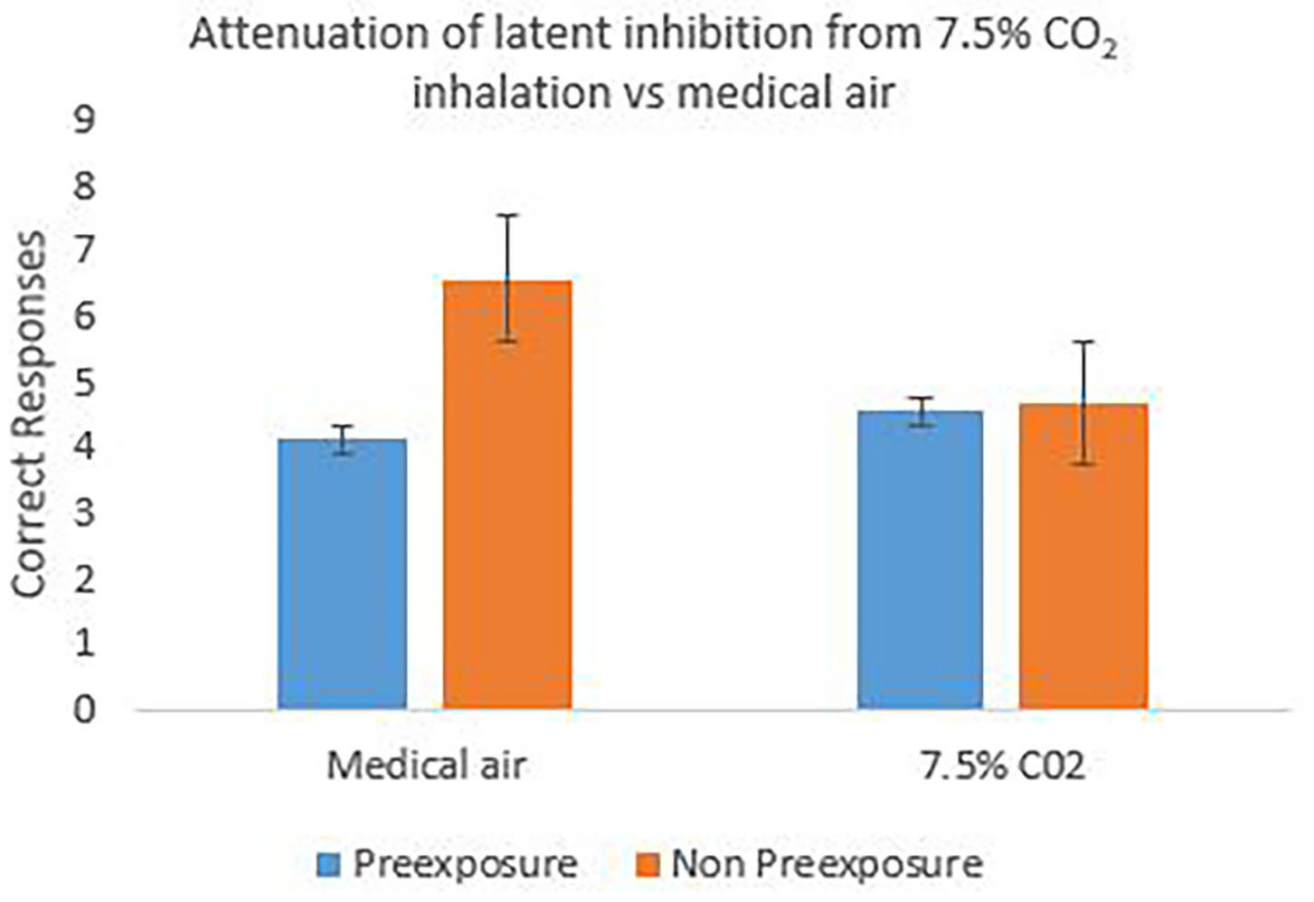
The mean number of correct responses to the target cued by preexposed stimuli and non-preexposed stimuli in the test stage of the latent inhibition task. Successful effect of latent inhibition is seen in the medical air condition, but attenuated or reversed during CO_2_ inhalation. Error bars are 1± within-subject standard error of the mean [see (45)].

Figure 3 shows the group mean of individual correct responses to the target (X or Z) across the 20 test trials with the preexposed and non-preexposed stimuli. For the medical air condition, it can be seen that correct responses were higher for the non-preexposed than the preexposed stimulus trials, illustrating a potential effect of latent inhibition. In the CO_2_ condition, by contrast, the amount of correct responses to both preexposed and non-preexposed stimuli appear relatively equal, indicating an absence of latent inhibition. This impression was confirmed with pre-planned comparisons revealing a significant effect of stimulus (preexposed vs. non-preexposed) only in the medical air condition, *F*_(1, 29)_ = 5.805, *p* = 0.023, partial η^2^ = 0.167, indicating the presence of latent inhibition. There was no significant effect of stimulus in the CO_2_ condition *F*_(1, 29)_ = 0.011, *p* = 0.919, indicating the absence of this effect (see Figure 3) in this sample of participants. There was however no overall main effect of stimulus *F*_(1, 29)_ = 2.690, *p* = 0.112 or of gas using a 2 (stimulus: preexposed, non-preexposed) × 2 (gas: CO_2_, medical air) repeated measures ANOVA but the overall 2-way interaction between stimulus × gas approached significance *F*_(1, 29)_ = 3.554, *p* = 0.069, partial η^2^ = 0.109.

### Discussion

Experiment 1 was successful in using 7.5% CO_2_ inhalation vs. medical air inhalation to induce state anxiety with results were in the anticipated direction: state anxiety measured by the STICSA (42) was significantly higher following CO_2_ inhalation with a large Cohen's d effect size. In addition, negative affect as measured by the PANAS (43), heart rate, systolic and diastolic blood pressure were all significantly higher following the inhalation of CO_2_, with generally large effect sizes. The validity of this manipulation to induce state anxiety is in line with previous research findings [see (38)].

Using both reaction time and correct response data, the results indicated that an effect of latent inhibition (faster/better learning to the non-preexposed stimuli compared to the preexposed stimuli) was only observable during the inhalations of medical air. During the 7.5% CO_2_ inhalations, the effect of latent inhibition was absent, which was particularly prominent when correct responses were used as the dependent variable. Interestingly, the absence of the latent inhibition effect in the CO_2_ condition seems to be primarily driven by a reduction in learning to the non-preexposed stimulus, indicating an observation of an induced learning deficit by CO_2_ exposure. The lack of overall interaction however between latent inhibition and gas condition is potentially due to a lack of power, as the sample size of the current study was relatively small. To increase the power of the study e.g., to 95%, we recommend the use of *N* = 40 in future studies to obtain a moderate effect size of *dz* = 0.6 at an alpha level of 5%. The direction of the current results nevertheless provide support for the Experiment 1 hypothesis and existing research that reports an absence and/or attenuation of latent inhibition under state anxiety, and by extension, augmented dopaminergic conditions relevant to schizophrenia [e.g., (10, 18–21)].

## Experiment 2: Effects of Nicotine on Latent Inhibition in Non-Smokers

### Materials and Methods

#### Design

To assess the sensitivity of latent inhibition to a pro-cognitive pharmacological manipulation, Experiment 2 evaluated latent inhibition in healthy non-smoking volunteers who received a 2 mg dose of nicotine or placebo in a single-blind crossover design with 2-day washout between treatment administrations.

#### Participants

Twenty non-smoking healthy volunteers were recruited from among members of the University of Bristol and the local community *via* email lists, poster and fllier advertisements and the Tobacco and Alcohol Research Group newsletter and website. Non-smokers were defined as not having smoked in the past 12 months, and not smoked more than 100 cigarettes in their lifetime. The exclusion criteria were age under 18 or over 50 years, pregnancy or breast feeding, recent use of prescribed or illicit drugs, uncorrected visual or hearing problems. Participants refrained from consuming alcohol for 24 h prior to the study day and were required to refrain from caffeine consumption on test days prior to assessments. Expired breath alcohol and carbon monoxide readings were taken, and participants were to be excluded if the readings were >0 or ≥10, respectively. No candidate participants had to be excluded from the research. The study was approved by the University of Bristol Faculty Of Science Research Ethics Committee. Sample size was determined based on a previous study of a similar nature (46).

#### Questionnaires and Latent Inhibition Task

A 12-item visual analog scale (VAS) was used to assess aversive effects of nicotine (nausea, dizziness, sweatiness, light-headed, nervous, headache, heart racing, indigestion, tight-throat, increased saliva, change in taste, fatigue), which relate to the most common side effects associated with acute nicotine administration reported in previous studies (47, 48). Additional questionnaires included the STICSA as measure of state and trait anxiety (42) and the O-LIFE as measure of schizotypy (44) to ensure baseline schizotypy scores were within normative range. The modified version of the Granger et al. (36) latent inhibition task was used, as described in Experiment 1.

#### Procedure

Eligible participants attended two sessions (minimum 2 days apart) at approximately the same time of day. After providing informed consent at the first testing session, further screening assessments were conducted and an expired CO test using a piCO smokelyser (Bedfont Scientific Ltd.) was used to rule out recent smoking. Baseline questionnaire measures (VAS, O-LIFE and STICSA) were then completed, after which participants were administered either 2 mg nicotine mouth spray or placebo (peppermint mouth spray, Boots UK). Treatment administration was single-blind and order of administration was counterbalanced across participants. Following administration, participants were required to sit quietly for 30 min to allow peak plasma nicotine levels to be reached. After which, the latent inhibition task was completed, followed by the self-report questionnaires. Prior to the second session, there was a washout period for a minimum of 2 days. The second session followed the same procedure as the first but delivered the alternative treatment (i.e., nicotine or placebo). At the end of the second session participants were debriefed and reimbursed £30.

### Results

#### Characteristics of Participants

The participants (*n* = 12; 60% female) were between 18 and 39 years of age (M = 23, SD = 4.6). STICSA trait baseline scores ranged between 22 and 42 (M = 31, SD = 6) and the sub-dimensions of O-LIFE between: Unusual Experiences (positive schizotypy); 0 and 15 (M = 5, SD = 4), Cognitive Disorganization; 0 and 18 (M = 8, SD = 6), Introvertive Anhedonia (negative schizotypy); 1 and 19 (M = 7, SD = 5) Impulsive Non-conformity; 1 and 14 (M = 6, SD = 3). O-LIFE scores were relatively comparable to normative values and those reported in previous studies (44) demonstrating baseline schizotypy scores representative of a healthy sample.

#### Subjective Effects (Nicotine vs. Placebo)

State anxiety (STICSA) and each of the VAS scores were higher after nicotine than after placebo (see Table 2), indicating that participants experienced the commonly experienced aversive effects of nicotine administration. At baseline, there were no significant differences between treatment groups (nicotine vs. placebo) for any of the subjective self-report measures (STICSA and VAS scores), derived using independent sample *t*-tests (all *p* > 0.07).

**Table 2:** State anxiety and subjective measures demonstrate anticipated aversive effects of 2 mg nicotine vs. placebo in non-smokers (paired *t*-test comparisons).

	**Mean difference (SD): nicotine vs. placebo**	**Effect Size (Cohen's d)**	***df***	**95% CI**	***p*-value**
STICSA state	3.25 (5.53)	0.54	19	0.66–5.84	0.017
Dizziness	23.60 (28.98)	1.11	19	10.04–37.16	0.002
Fatigue	1.85 (14.02)	0.08	19	−4.71–8.41	0.562
Headache	8.10 (20.26)	0.35	19	−1.38–17.58	0.090
Heart racing	11.00 (15.25)	0.69	19	3.86–18.14	0.004
Indigestion	2.45 (8.65)	2.44	19	−1.60–6.50	0.221
Nausea	13.90 (24.56)	0.74	19	2.41–25.39	0.020
Nervousness	7.90 (19.49)	0.54	19	−1.22–17.02	0.086
Salivation	7.65 (15.89)	0.38	19	0.21–15.09	0.044
Sweatiness	11.20 (19.47)	0.77	19	2.09–20.31	0.019
Taste	10.05 (20.75)	0.51	19	0.34–19.76	0.043
Throat-tightness	22.45 (31.41)	1.00	19	7.75–37.15	0.005

*STICSA, State-Trait Inventory for Cognitive and Somatic Anxiety*.

#### Latent Inhibition: Reaction Time

**Figure 4:**
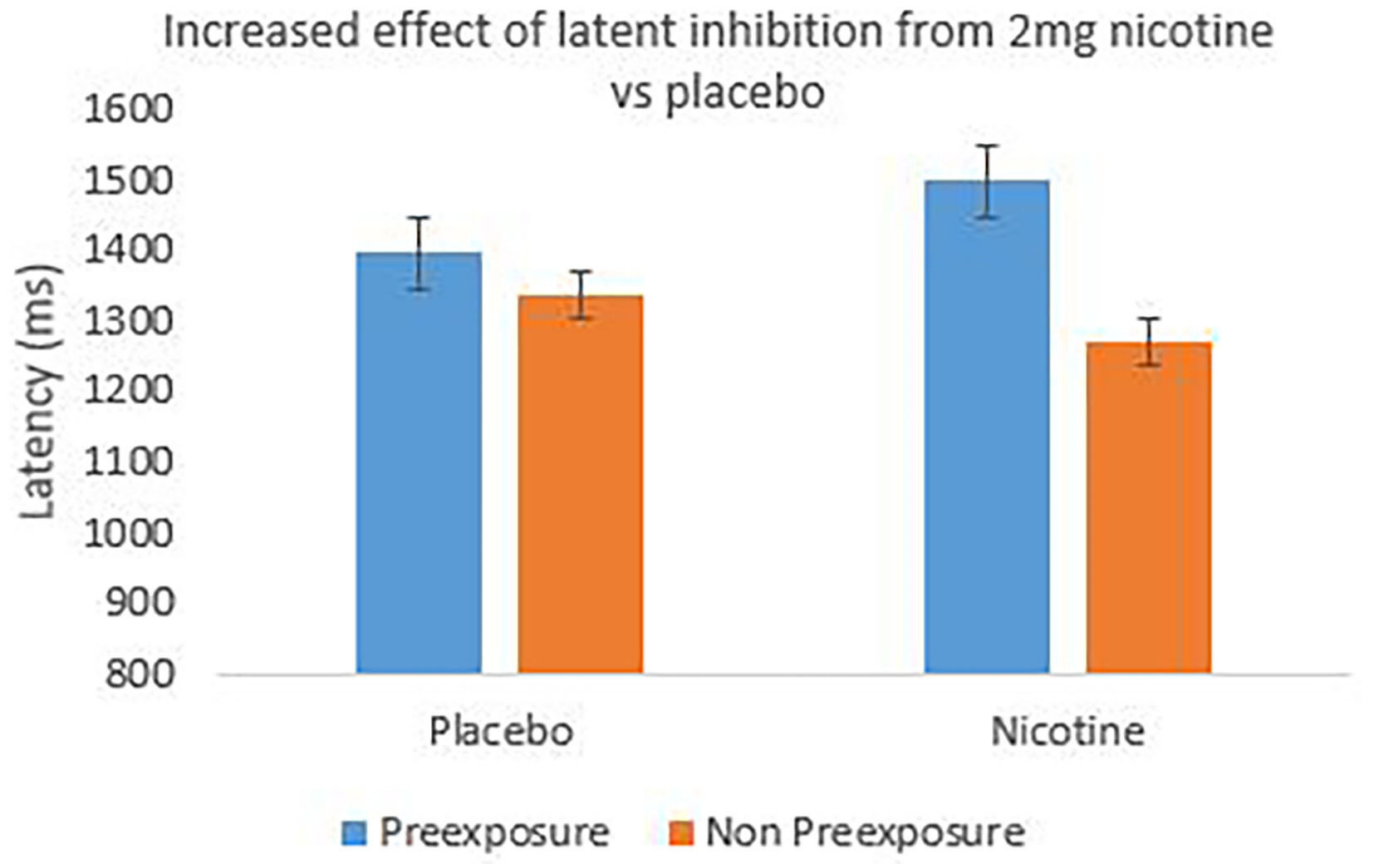
The mean reaction time to the target cued by preexposed stimuli and non-preexposed stimuli in the test stage of the latent inhibition task. A potentiation of latent inhibition is seen in the nicotine treatment arm which is reduced for the placebo arm. Error bars are 1± within-subject standard error of the mean [see (45)].

Figure 4 shows the group mean of individual median reaction times to the target (X or Z) across the 20 test trials with the preexposed and non-preexposed stimuli. For the nicotine condition, reaction times were faster during the non-preexposed than the preexposed stimulus trials, indicating an effect of latent inhibition compared to the placebo group. This impression was explored using a 2 (stimulus: preexposed, non-preexposed) × 2 (treatment: nicotine, placebo) repeated measures ANOVA on individual median reaction times, which revealed a significant main effect of stimulus *F*_(1, 19)_ = 6.246, *p* = 0.002, partial η^2^ = 0.247, indicating an overall effect of latent inhibition; there was no main effect of treatment (*F* < 1). Pre-planned comparisons revealed a significant effect of stimulus only in the nicotine treatment *F*_(1, 19)_ = 7.288, *p* = 0.014, partial η^2^ = 0.277, indicating an effect of latent inhibition. There was however no significant effect of stimulus in the placebo arm (*F* < 1), see Figure 4, indicating an absence/reduction of the effect compared to the nicotine treatment. The overall 2-way interaction (stimulus × treatment) was however not significant (*F* < 1).

#### Latent Inhibition: Correct Responses

**Figure 5:**
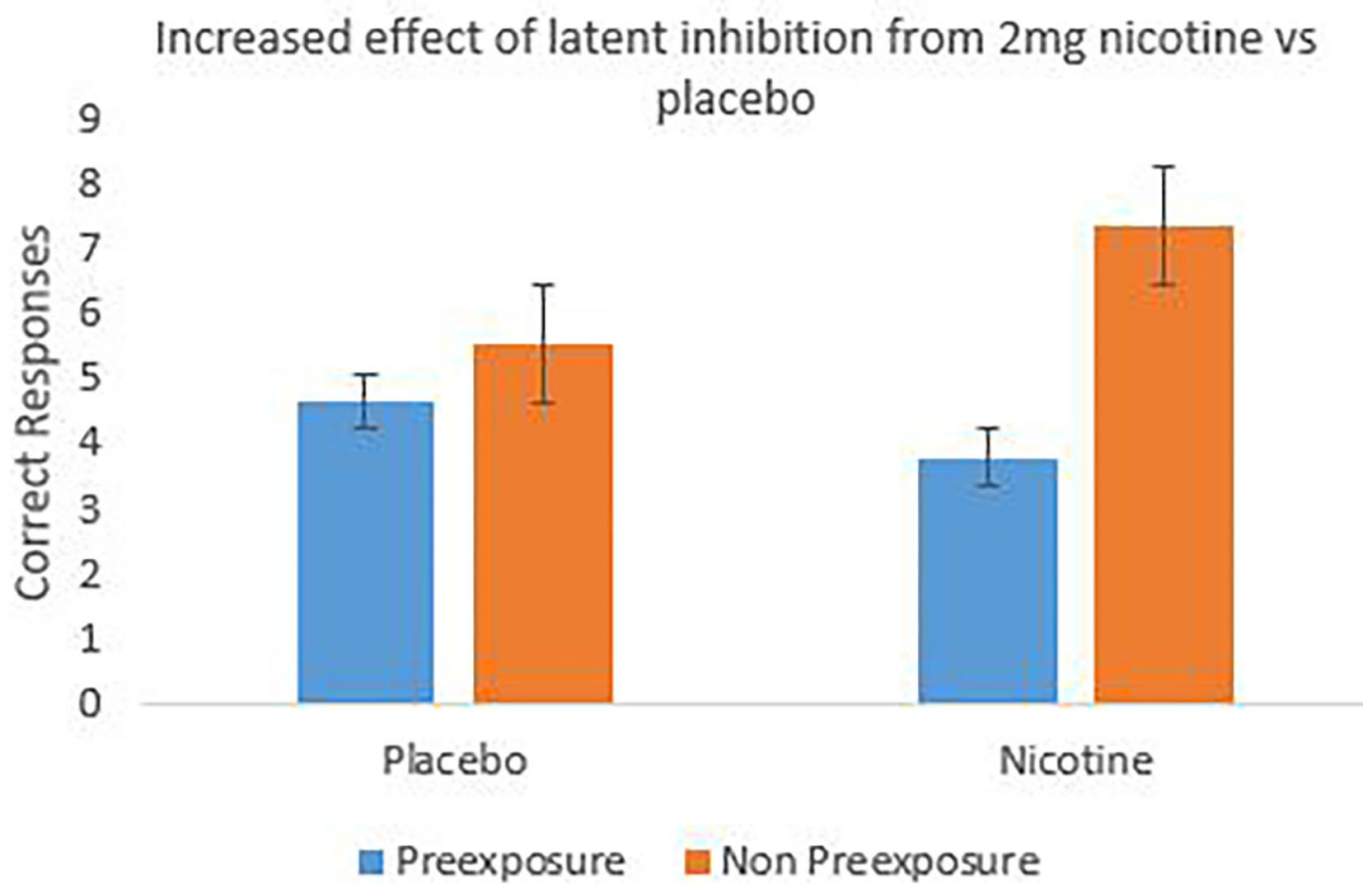
The mean number of correct responses to the target cued by preexposed stimuli and non-preexposed stimuli in the test stage of the latent inhibition task. A potentiation of latent inhibition is seen in the nicotine treatment arm which is reduced for the placebo arm. Error bars are 1± within-subject standard error of the mean [see (45)].

Figure 5 shows the group mean of individual correct responses to the target (X or Z) across the 20 test trials with the preexposed and non-preexposed stimuli. For the nicotine treatment arm, correct responses were higher for the non-preexposed than the preexposed stimulus trials, indicating an effect of latent inhibition that appears increased relative to the placebo treatment arm. This impression was explored using a 2 (stimulus: preexposed, non-preexposed) × 2 (treatment: nicotine, placebo) repeated measures ANOVA on individual correct responses, which revealed a significant main effect of stimulus *F*_(1, 19)_ = 7.563, *p* = 0.013, partial η^2^ = 0.285 indicating an overall effect of latent inhibition; there was no significant main effect of treatment (*F* < 1). Pre-planned comparisons revealed a significant effect of stimulus only in the nicotine treatment arm, *F*_(1, 19)_ = 6.717, *p* = 0.018, partial η^2^ = 0.261, indicating an effect of latent inhibition. There was however no significant effect of stimulus in the placebo arm (*F* < 1), see Figure 5. The overall 2-way interaction (stimulus × treatment) was however not significant (*F* < 1).

### Discussion

This experiment confirmed that nicotine (vs. placebo) induced the commonly experienced aversive effects in non-smokers [cf. (49)], in particular, state anxiety, racing heart, nervousness, sweatiness, and throat-tightness. Both reaction time and correct response data confirmed an overall effect of latent inhibition. Nicotine treatment appeared to produce a greater degree of latent inhibition than the placebo arm (see Figure 5 in particular) indicated by the significant effect of stimulus relative to nicotine administration but not placebo. Whilst the effect of stimulus in the placebo arm was not significant, the anticipated direction of effect for latent inhibition was observable (in particular more correct responses to the non-preexposed stimuli, compared to the preexposed stimuli; Figure 5). This lack of/small demonstration of latent inhibition in the placebo arm could potentially be a result of participant's anticipation of receiving nicotine, generating a compensatory response that is agonistic to the normal effect of nicotine [see e.g., (50)]. In line with this, the anticipatory effect would then also presumably be present in the nicotine treatment condition, but overcome by the pharmacological effect of nicotine itself, as illustrated by the presence of latent inhibition. The statistical exploration of this in the current study e.g., by exploring the order effects of treatment administration on latent inhibition is not however attainable due to restrictions on sample size. To increase the power of the study e.g., to 95%, we recommend the use of *N* = 40 in future studies to obtain a moderate effect size of *dz* = 0.6 at an alpha level of 5%. Nevertheless, the current finding which illustrates an observable effect of latent inhibition from nicotine administration, compared to placebo, provides support for existing research [e.g., (31)]. It would be of interest for future research to explore differences in latent inhibition to e.g., 2 vs. 4 mg of nicotine to establish dose sensitivity of the latent inhibition effect. In addition, to further understand the effects of nicotine on latent inhibition, a larger future research study could recruit smokers and non-smokers to evaluate whether a reduced effect of latent inhibition potentiation by nicotine is observed in those who already smoke cigarettes, compared to those who do not [cf. (51)].

#### General Discussion

Inhalation of 7.5% CO_2_ raised self-report and physiological measures of anxiety and impaired latent inhibition compared to medical air control; whereas administration of nicotine demonstrated an increased effect of latent inhibition, compared to placebo control. Given supporting evidence that state anxiety increases dopamine (39, 40), the aim of Experiment 1 was to explore the sensitivity of the modified latent inhibition task (36) to an in-direct model relevant to psychosis (positive symptoms associated with schizophrenia) by using the 7.5% CO_2_ challenge as a way to stimulate dopamine release *via* induction of state anxiety. In contrast, Experiment 2 aimed to explore the sensitivity of latent inhibition to a pro-cognitive model relevant to the treatment of cognitive impairment associated with schizophrenia by examining the effect of nicotine administration on latent inhibition vs. placebo. The results from these initial experiments suggest latent inhibition warrants further investigation as a potential biomarker for schizophrenia [see also (24)].

Given the sensitivity of latent inhibition to dopaminergic manipulations as seen from existing research [see (10)], and by corollary underlying dopaminergic perturbations observed in psychosis patients (9), further studies should assess whether latent inhibition can be used as a tool to help identify patients and also accelerate or rationalize treatment strategies for patients with psychotic disorders to support decision making. With no biomarker currently available to identify, for example, individuals at ultra-high risk (UHR) for developing psychosis, a means to do so would allow anti-psychotic treatment to be initiated at an earlier stage to reduce the risk of conversion to a full-blown state of psychosis. Currently, treatment for psychosis is not initiated until the first full episode of the disorder emerges (52), and is thus rarely (if at all) provided to UHR individuals. Given existing research supporting the sensitivity of latent inhibition, it has the potential, with further clinical validation, to act as a surrogate marker to detect underlying neurotransmitter perturbations and provide a non-invasive proxy measure of e.g., hyper-dopaminergic state to identify which individuals would, along biological lines, be suited to receiving a dopamine blocker (the mainstream anti-psychotic treatment) to remediate psychosis, or a non-dopaminergic alternative. Considering around one third of patients are also classified as treatment resistant [see (53)], it is a major clinical need to identify ways for patients to be fast-tracked to an appropriate treatment, ideally at initial diagnosis depending upon their neurobiology. Experimental investigations should continue to focus on this in future research, particularly as specialist clinical services are well-placed to benefit from novel means for better identification and/or early treatment options for affected individuals.

The effect of latent inhibition by nicotine administration compared to placebo observed in Experiment 2, provides encouraging support for existing research demonstrating a potentiation of latent inhibition in smokers compared to non-smokers (32) and for preclinical findings that demonstrate demonstrating pro-cognitive effects of an α7-nAChR partial agonist, SSR180711 using latent inhibition as a model to demonstrate treatment efficacy [see (33)]. Given the sensitivity of latent inhibition to cholinergic manipulations and associated neurobiological disruptions, future research should investigate the sensitivity of latent inhibition as a stratification tool to identify the sub-population of patients with schizophrenia that could benefit from pro-cognitive treatment with a α7-nAChR agonist. Despite the biological complexity and heterogeneity of schizophrenia, inclusion criterion for previous clinical trials investigating these potentially pro-cognitive drugs have relied on subjective diagnoses and self-report measures (i.e., the Diagnostic and Statistical Manual of Mental Disorders, 5th Edition: DSM-5). Since DSM-5 criteria neither determine the presence of cognitive impairments cognitive ability nor classify according to underlying neurobiological abnormalities, it is not surprising that these drugs have failed to universally improve cognition among such a heterogeneous group. To date, 87 novel agents have been unsuccessfully trialed for cognitive impairment associated with schizophrenia [see (54)]: a tool to enhance the prediction of treatment efficacy for a core area of schizophrenia where no treatments currently exist has the potential to greatly improve the chances of an effective drug becoming available.

### Conclusions

The experiments reported here provide initial research findings that support the potential utility and sensitivity of latent inhibition to relevant manipulations which underpin key neurobiological dysfunctions and symptoms associated with schizophrenia; a tool that is sensitive to these neurobiological states and associated treatment-induced changes holds potential to advance schizophrenia research. Latent inhibition holds potential promise as a biomarker/stratification tool for use in both clinical practice and clinical development for patients that are in need of improved means of illness detection, and improved efficacy of treatment options and outcomes.

## Data Availability Statement

The raw data supporting the conclusions of this article will be made available by the authors, without undue reservation.

## Ethics Statement

The studies involving human participants were reviewed and approved by University of Bristol, Faculty of Science Research Ethics Committee. The patients/participants provided their written informed consent to participate in this study.

## Author Contributions

KG and JB designed the study protocol. KG drafted the manuscript. JF carried out recruitment and experimental testing with oversight and project management from AA. All authors critically reviewed and approved the manuscript prior to its submission for publication.

## Conflict of Interest

KG, SC, and JB are employees of Cambridge Cognition Ltd. KG, SC, and JB are also employees of Monument Therapeutics Ltd. The remaining authors declare that the research was conducted in the absence of any commercial or financial relationships that could be construed as a potential conflict of interest.

## References

[1] Lubow RE. Latent inhibition. Psychol Bull. (1973) 79:398–407. 10.1037/h0034425 4575029

[2] Hall G Honey RC. Contextual effects in conditioning, latent inhibition, and habituation: Associative and retrieval functions of contextual cues. J Exp Psychol Anim Behav Processes. (1989) 15:232–41. 10.1037/0097-7403.15.3.232

[3] Moser PC Hitchcock JM Lister S Moran PM. The pharmacology of latent inhibition as an animal model of schizophrenia. Behav Brain Res. (2000) 33:275–307. 10.1016/S0165-0173(00)00026-6 11011070

[4] Arad M Weiner I. Disruption of latent inhibition induced by ovariectomy can be reversed by estradiol and clozapine as well as by co-administration of haloperidol with estradiol but not by haloperidol alone. Psychopharmacology. (2009) 206:731–40. 10.1007/s00213-009-1464-0 19169876

[5] Lubow RE Moore AU. Latent inhibition: the effect of nonreinforced preexposure to the conditional stimulus. J Comp Physiol Psychol. (1959) 52:415–9. 10.1037/h0046700 14418647

[6] Lubow RE Gewirtz JC. Latent inhibition in humans: data, theory, and implications for schizophrenia. Psychol Bull. (1995) 117:87–103. 10.1037/0033-2909.117.1.87 7870865

[7] Mackintosh NJ. Blocking of conditioned suppression: role of the first compound trial. J Exp Psychol Anim Behav Processes. (1975) 1:335–45. 10.1037/0097-7403.1.4.335 1202140

[8] Pearce JM Hall G. A model for pavlovian learning: variations in the effectiveness of conditioned but not of unconditioned stimuli. Psychol Rev. (1980) 87:532–52. 10.1037/0033-295X.87.6.532 7443916

[9] Lubow RE Weiner I. Issues in latent inhibition research and theory: an overview. In: Lubow RE Weiner I editors. Latent Inhibition: Cognition, Neuroscience, and Applications to Schizophrenia. New York, NY: Cambridge University Press (2010). p. 531–557.

[10] Weiner I. The “two-headed” latent inhibition model of schizophrenia: modeling positive and negative symptoms and their treatment. Psychopharmacology. (2003) 169:257–97. 10.1007/s00213-002-1313-x 12601500

[11] Weiner I Arad M. Using the pharmacology of latent inhibition to model domains of pathology in schizophrenia and their treatment. Behav Brain Res. (2009) 204:369–86. 10.1016/j.bbr.2009.05.004 19433114

[12] Abi-Dargham A Gil R Krystal J Baldwin RM Seibyl JP Bowers M. Increased striatal dopamine transmission in schizophrenia: confirmation in a second cohort. Am J Psychiatry. (1988) 155:761–7. 9619147 10.1176/ajp.155.6.761

[13] Bramness JG Rognli EB. Psychosis induced by amphetamines. Curr Opin Psychiatry. (2016) 29:236–41. 10.1097/YCO.0000000000000254 27175554

[14] Solomon PR Crider A Winkelman JW Turi A Kramer RM Kaplam LJ. Disrupted latent inhibition in the rat with chronic amphetamine or haloperidol-induced supersensitivity: relationship to schizophrenic attention disorder. Biol Psychiatry. (1981) 16:529–37. 7196265

[15] Weiner I Lubow RE Feldon J. Disruption of latent inhibition by acute administration of low doses of amphetamine. Pharmacol Biochem Behav. (1988) 30:871–8. 10.1016/0091-3057(88)90113-X 3227035

[16] Amador M Dani JA. MK-801 inhibition of nicotinic acetylcholine receptor channels. Synapse. (1991) 7:207–15. 10.1002/syn.890070305 1715611

[17] Lewis MC Gould TJ. Latent inhibition of cued fear conditioning: an NMDA receptor-dependent process that can be established in the presence of anisomycin. Eur J Neurosci. (2004) 20:818–26. 10.1111/j.1460-9568.2004.03531.x 15255992

[18] Baruch I Hemsley DR Gray JA. Differential performance of acute and chronic schizophrenics in a latent inhibition task. J Nerv Ment Dis. (1988) 176:598–606. 10.1097/00005053-198810000-00004 2903219

[19] Gray NS Hemsley DR Gray JA. Abolition of latent inhibition in acute but not chronic schizophrenics. Neurol Psychiatry Brain Res. (1992) 1:83–9.

[20] Rascle C Mazas O Vaiva G Tournant M Raybois O Goudemand M. Clinical features of latent inhibition in schizophrenia. Schizophr Res. (2001) 51:149–61. 10.1016/S0920-9964(00)00162-6 11518635

[21] Vaitl D Lipp O Bauer U Schuler G Stark R Zimmermann M. Latent inhibition and schizophrenia: Pavlovian conditioning of autonomic responses. Schizophr Res. (2002) 55:147–58. 10.1016/S0920-9964(01)00250-X 11955974

[22] Cohen E Sereni N Kaplan O Weizman A Kikinzon L Weiner I. The relation between latent inhibition and symptom-types in young schizophrenics. Behav Brain Res. (2004) 149:113–22. 10.1016/S0166-4328(03)00221-3 15129775

[23] Gal G Barnea Y Biran L Mendlovic S Gedi T Halavy M. Enhancement of latent inhibition in patients with chronic schizophrenia. Behav Brain Res. (2009) 197:1–8. 10.1016/j.bbr.2008.08.023 18793680

[24] Granger KT Talwar A Barnett JH. Latent inhibition and its potential as a biomarker for schizophrenia. Biomark Neuropsychiatry. (2020) 3:100025. 10.1016/j.bionps.2020.100025

[25] Marder SR. The NIMH-MATRICS project for developing cognition-enhancing agents for schizophrenia. Dialogues Clin Neurosci. (2006) 8:109–13. 10.31887/DCNS.2006.8.1/smarder 16640121 PMC3181758

[26] Hill SK Bishop JR Palumbo D Sweeney JA. Effect of second-generation antipsychotics on cognition: current issues and future challenges. Expert Rev Neurother. (2010) 10:43–57. 10.1586/ern.09.143 20021320 PMC2879261

[27] MacKenzie NE. Antipsychotics, metabolic adverse effects, and cognitive function in schizophrenia. Front Psychiatry. (2018) 9:622. 10.3389/fpsyt.2018.00622 30568606 PMC6290646

[28] Kumari V Postma P. Nicotine use in schizophrenia: the self medication hypotheses. Neurosci Biobehav Rev. (2005) 29:21–34. 10.1016/j.neubiorev.2005.02.006 15964073

[29] Wootton R Richmond R Stuijfzand B Lawn R Sallis H Taylor G. Evidence for causal effects of lifetime smoking on risk for depression and schizophrenia: a Mendelian randomisation study. Psychol Med. (2019) 50:1−9. 10.1017/S0033291719002678 31689377 PMC7610182

[30] Smucny J Tregellas JR. Targeting neuronal dysfunction in schizophrenia with nicotine: evidence from neurophysiology to neuroimaging. J Psychopharmacol. (2017) 3:801–11. 10.1177/0269881117705071 28441884 PMC5963521

[31] Thornton JC Dawe S Lee C Capstick C Corr PJ Cotter P. Effects of nicotine and amphetamine on latent inhibition in human subjects. Psychopharmacology. (1996) 127:164–73. 10.1007/BF02805990 8888383

[32] Della Casa V Weiner I Feldon J. Effects of smoking status and schizotypy on latent inhibition. J Psychopharmacol. (1999) 13:45–57. 10.1177/026988119901300106 10221359

[33] Barak S Arad M De Levie A Black MD Griebel G Weiner I. Pro-cognitive and antipsychotic efficacy of the A7 nicotinic partial agonist SSR180711 in pharmacological and neurodevelopmental latent inhibition models of schizophrenia. Neuropsychopharmacology. (2009) 34:1753–63. 10.1038/npp.2008.232 19158670

[34] Shiina A Shirayama Y Niitsu T Hashimoto T Yoshida T Hasegawa T. A randomised, double-blind, placebo-controlled trial of tropisetron in patients with schizophrenia. Ann Gen Psychiatry. (2010) 9:1–10. 10.1186/1744-859X-9-27 20573264 PMC2901366

[35] Jones LA Hills PJ Dick KM Jones SP Bright P. Cognitive mechanisms associated with auditory sensory gating. Brain Cogn. (2016) 102:33–45. 10.1016/j.bandc.2015.12.005 26716891 PMC4727785

[36] Granger KT Moran PM Buckley MG Haselgrove M. Enhanced latent inhibition in high schizotypy individuals. Pers Individ Dif. (2016) 91:31–9. 10.1016/j.paid.2015.11.04022579971

[37] Garner M Attwood A Baldwin DS James A Munafo M. Inhalation of 7.5% carbon dioxide increases threat processing in humans. Neuropsychopharmacology. (2011) 36:1557–62. 10.1038/npp.2011.15 21490591 PMC3138667

[38] Easey KE Catling JC Kent C Crouch C Jackson S Munafò MR. State anxiety and information processing: a 7.5% carbon dioxide challenge study. Psychon Bull Rev. (2018) 25:732–8. 10.3758/s13423-017-1413-6 29392633 PMC5902516

[39] Mizrahi R Addington R Rusjan I Ng A Boileau I Pruessner JC. Increased stress-induced dopamine release in psychosis. Biol Psychiatry. (2012) 71:561–7. 10.1016/j.biopsych.2011.10.009 22133268

[40] Nagano-Saito A Dagher A Booij L Gravel P Welfeld K Casey KF. Stress-induced dopamine release in human medial prefrontal cortex-−18F-fallypride/PET study in healthy volunteers. Synapse. (2013) 67:821–30. 10.1002/syn.21700 23939822

[41] Sheehan DV Lecrubier Y Sheehan KH Amorim P Janavs J Weiller E. The mini-international neuropsychiatric interview (M.I.N.I.): the development and validation of a structured diagnostic psychiatric interview for DSM-IV and ICD-10. J Clin Psychiatry. (1998) 59:22–33. 10.1037/t18597-000 9881538

[42] Ree MJ French D MacLeod C Locke V. Distinguishing cognitive and somatic dimensions of state and trait anxiety: development and validation of the state-trait inventory for cognitive and somatic anxiety (STICSA). Behav Cogn Psychother. (2008) 36:313–32. 10.1017/S1352465808004232

[43] Watson D Clark LA Tellegen A. Development and validation of brief measures of positive and negative affect: the PANAS scales. J Pers Soc Psychol. (1988) 54:1063–70. 10.1037/0022-3514.54.6.1063 3397865

[44] Mason O Claridge G Jackson M. New scales for the assessment of schizotypy. Personal Individ Differ. (1995) 18:7–13. 10.1016/0191-8869(94)00132-C

[45] Cousineau D. Confidence intervals in within-subject designs: a simpler solution to Loftus and Masson's method. Tutorial Quantitative Methods Psychol. (2005) 1:4–45. 10.20982/tqmp.01.1.p042

[46] Griesar WS Zajdel DP Oken BS. Nicotine effects on alertness and spatial attention in non-smokers. Nicotine Tobacco Res. (2002) 4:185–94. 10.1080/14622200210123617 12028851

[47] Blank M Sams C Weaver MF Eissenberg T. Nicotine delivery, cardiovascular profile, and subjective effects of an oral tobacco product for smokers. Nicotine Tob Res. (2008) 10:417–21. 10.1080/14622200801901880 18324559 PMC3207995

[48] Shahab L McEwen A West R. Acceptability and effectiveness for withdrawal symptom relief of a novel oral nicotine delivery device: a randomised cross-over trial. Psychopharmacology. (2011) 216:187–96. 10.1007/s00213-011-2204-9 21318563

[49] Adams S Attwood A Munafò M. Effects of nicotine and nicotine expectancy on attentional bias for emotional stimuli. Nico. Tob Res. (2015) 17:697–703. 10.1093/ntr/ntu219 25335948 PMC5011420

[50] Siegel S Baptista MAS Kim JA McDonald RV Weise-Kelly L. Pavlovian psychopharmacology: the associative basis of tolerance. Exp Clin Psychopharmacol. (2000) 8:276-93. 10.1037/1064-1297.8.3.276 10975617

[51] Tregellas JR Wylie KP. Alpha 7 nicotinic receptors as therapeutic targets in schizophrenia. Nicotine Tob Res. (2019) 21:349–56. 10.1093/ntr/nty034 30137618 PMC6379034

[52] Fusar-Poli P Bonoldi I Yung AR Borgwardt S Kempton MJ Valmaggia L. Predicting psychosis: meta-analysis of transition outcomes in individuals at high clinical risk. Arch Gen Psychiatry. (2012) 69:220–9. 10.1001/archgenpsychiatry.2011.1472 22393215

[53] Lally J MacCabe JH. Antipsychotic medication in schizophrenia: a review. Br Med Bull. (2015) 14:169–79. 10.1093/bmb/ldv017 25957394

[54] Cotter J Barnett JH Granger K. The use of cognitive screening in pharmacotherapy trials for cognitive impairment associated with schizophrenia. Front Psychiatry. (2019) 10:648. 10.3389/fpsyt.2019.00648 31551837 PMC6743013

